# Family Cohesion, Adaptability, and Oral Health in Indigenous Malaysian Children: A Pilot Study

**DOI:** 10.7759/cureus.92620

**Published:** 2025-09-18

**Authors:** Nurul Fahizha Fahimi, Norashikin Yusof, Budi Aslinie Md Sabri

**Affiliations:** 1 Centre of Population Oral Health and Clinical Prevention, Faculty of Dentistry, Universiti Teknologi MARA, Sungai Buloh, MYS

**Keywords:** children, family cohesion, oral health, orang asli, pilot study

## Abstract

Aim: Indigenous children in Malaysia face high oral disease burdens, yet family dynamics influencing their oral health remain poorly understood. They are considered an economically and socially marginalized population. Despite efforts to enhance their quality of life, they have a comparatively worse health state when compared to the overall population. Family cohesion is the degree of emotional intimacy among individuals within a family unit. It was highlighted as a factor influencing overall health. This pilot study aimed to examine the associations between family cohesion, family adaptability, and maternal oral health with the oral health status of Orang Asli children aged 5-12 years in Kampung Pelawan, Perak.

Material and methods: Data collection was conducted after ethics approval. A total of 30 dyads of a parent and a child between the ages of 5 and 12 years old from an Orang Asli village in Perak were randomly chosen as participants. After giving consent, oral examinations were conducted, and caries were charted according to the decayed, missing, and filled teeth in permanent dentition/decayed and filled teeth in primary dentition (DMFT/dft) index. Family cohesion and adaptability were measured using the Family Adaptability and Cohesion Evaluation Scale-III (FACES-III).

Results: The participants consist of 30 mothers with a mean age of 35.67. The prevalence of caries among parents was 93.3% (mean DMFT=5). The prevalence of caries in primary teeth among children is 46.7% (mean dft=3.17). The family cohesion levels are disengaged (36.7%) and connected (33.3%), while 76.7% are chaotic. Mothers aged less than 40 years (RR=4.72; 95% CI:1.02-21.9) and a chaotic type of family adaptability (RR=6.85; 95% CI:1.09-42.89) were associated with higher caries in children.

Conclusions: This pilot study suggests that younger maternal age and chaotic family adaptability are significant predictors of caries in primary teeth among Orang Asli children, while family cohesion did not show a direct association. Family-centered and culturally tailored interventions are needed to strengthen family functioning and address parental factors, with larger studies required to confirm these associations.

## Introduction

Oral health is a key component of overall health and quality of life [1]. However, oral diseases such as dental caries remain highly prevalent and disproportionately affect disadvantaged populations. In Malaysia, the Orang Asli (OA), the indigenous peoples of Peninsular Malaysia, experience significant oral health disparities compared to the general population, driven by socioeconomic disadvantage, geographic isolation, and limited access to dental services [2]. National and local studies have reported high rates of untreated dental caries, periodontal disease, and tooth loss among OA children and adults [3,4]. Despite various government initiatives, improvements in OA oral health have been slow. This persistent inequality underscores the need for targeted and culturally sensitive strategies to address the oral health needs of this community.

Family environment is recognized as a critical determinant of health-related behaviors [5]. In particular, family cohesion (emotional bonding among family members) [6] and family adaptability (capacity to adjust roles and structures in response to change) [7] influence oral hygiene practices, dietary habits [8,9], and dental attendance patterns [10,11]. Evidence from other populations suggests that stronger family cohesion is associated with better oral health outcomes in children [12], but little is known about this relationship in OA communities [13]. Given the unique cultural context of OA communities, understanding the influence of family cohesion and adaptability on oral health may help inform tailored interventions. Therefore, the aim of this pilot study was to examine the association between family cohesion and adaptability and the oral health status of Orang Asli children aged 5 to 12 years old in Kampung Pelawan, Perak, as well as the influence of mothers’ oral health on children’s caries outcomes.

## Materials and methods

Ethical clearance

This study received approval from the Research Ethics Committee of the Research Management Centre, Universiti Teknologi MARA (UiTM), Malaysia (REC/08/2023(PG/FB/15)), and the Department of Orang Asli Development, Ministry of Rural and Regional Development, Malaysia (DOADM).

Study design and setting

This study is a cross-sectional study. The data collection was conducted from 25 to 30 June 2024 in Kampung Pelawan, Langkap, Perak, Malaysia. This village was chosen based on the systematic random sampling performed on the lists of Orang Asli villages provided by the DOADM [14]. To put it into context, Kampung Pelawan, a village populated by Orang Asli of Semai ethnicity, is located 79 kilometers south of Ipoh, Perak, Malaysia. With a total population of 634 people, the primary source of income is agriculture [15].

Study population and sample

Data collection was conducted after receiving approval from the DOADM with the help of "Tok Batin," the head of the village. The sample size for this pilot study was 30 [16]. Within the selected village, purposive sampling was used to identify eligible households, as confirmed by the Tok Batin. Only households with children aged 5 to 12 years and a parent able to converse in Bahasa Melayu were invited to participate. Contrarily, children who do not live with their mothers or live in institutions were excluded from this study.

Data collection procedures

Households identified as eligible were approached and informed about the study. Once a household was selected, it was approached and informed about the research details. If a household declined to participate, the next eligible household was approached until the target sample size of 30 dyads was achieved. In households with more than one eligible child, the parent was asked to select one child to represent the family. Recruitment continued until no additional eligible households remained. Validated questionnaires were administered to the mothers to collect data on their sociodemographic profiles and family cohesion levels. Dental examinations were conducted on dyads by a dentist with more than 10 years of experience. The examination was conducted either inside the participant's house or outside at the patio or a small hut. The participant was seated comfortably on any available household chair. A portable light and sterile examination sets were used to aid the process. 

Questionnaire and validation process

The questionnaires on family cohesion were developed based on the Family Adaptability and Cohesion Evaluation Scale (FACES-III) [17], an open-access instrument, which was used to assess family cohesion, and no permission is required for its use. The complete questionnaire is provided in Appendix 1. The questionnaire consists of 10 even-numbered questions measuring family cohesion and 10 odd-numbered questions measuring family adaptability. FACES-III was used as an instrument in this research, as it is available in open access and is frequently used in this country [7]. This study utilized the Family Adaptability and Cohesion Evaluation Scale III (FACES-III) developed by Olson, Portner, and Lavee (1985) [18]. Although FACES-III is no longer commercially available and has since been replaced by FACES-IV, we used FACES-III because it was widely validated at the time of this study. The original source has been cited accordingly. Following editorial advice, the authorized distributor of Dr. David Olson’s family assessment tools was contacted and informed that FACES-III is no longer sold or licensed, as it has been superseded by FACES-IV. The questions were then translated from English to Bahasa Melayu and then back-translated to ensure they retained the exact details of the original version [19]. The expert committee members, who consisted of two dental public health specialists, established a consensus on every issue and finalized the questionnaire in Bahasa Melayu. 

To ensure cultural appropriateness of the questionnaire, this pilot study also functioned as a validation exercise. After administering the questionnaire, feedback from participating mothers was systematically collected. Participants were asked to comment on the clarity and relevance of items. Their feedback was then analyzed thematically to identify cultural or linguistic issues. Several items required modification for better comprehension. For example, “bacteria” was replaced with the more familiar Malay term “kuman”; “plaque” was replaced with “sisa makanan” (food debris); and additional examples, such as charcoal and sand, were added to oral hygiene practice questions. For family cohesion items, probing questions were suggested to contextualize concepts such as household rules and parental punishment. These refinements improved cultural fit and ensured that the questionnaire would be better understood in the larger main study.

Examiner calibration

To ensure standardization of dental charting and examination, both inter- and intra-examiner reliability tests were conducted during the pilot study. For inter-examiner reliability, the study examiner, who had prior training with pediatric dental specialists, was calibrated against an expert with more than 30 years of experience. Both examiners independently assessed 15 dyads (30 individuals). Agreement was quantified using Cohen’s kappa statistic, which yielded a score of 0.85, indicating almost perfect agreement. For intra-examiner reliability, duplicate examinations were conducted by the same examiner on 15 dyads during the pilot study, with Cohen’s kappa again demonstrating strong consistency. These results confirm the reliability of the examiner’s clinical assessments.

Independent variables

The sociodemographic characteristics for this study included gender, age, ethnicity, sub-ethnicity, religion, and household wealth. Family cohesion is classified (in decreasing order) as enmeshed (high level of family dependence), connected (moderate family independence), separated (considerable family independence), or disengaged (very high level of family independence) [20]. Family adaptability is classified (in decreasing order) as chaotic (very high level of flexibility), flexible (moderate to high family flexibility), structured (low to moderate family flexibility), or rigid (very low level of flexibility) [20].

Dependent variables

During the examination, decayed, missing, and filled teeth in permanent dentition/decayed and filled teeth in primary dentition (DMFT/dft) index [21] was used to collect data on the caries experience. Participants were then categorized under caries-free (DMFT or dft=0), low (DMFT or dft=1 or 2), and high caries (DMFT or dft≥3) [22]. At the same time, the Silness and Löe Plaque Score was used to collect data on the oral hygiene of the participants [23]. Plaque disclosing gel was used to assist in the plaque charting of the surfaces for six index teeth: 16 (buccal), 12/52 (labial), 24/64 (buccal), 36 (lingual), 32/72 (lingual), and 44/84 (lingual). The tooth surface was rated on a scale from 0 to 3. The scores from the surfaces were summed and divided by the number of inspected teeth to calculate the individual's plaque index [24]. The participants were then categorized based on the score: excellent (score=0), good (0.1-0.9), fair (1.0-1.9), and poor (2.0-3.0).

Data management and statistical analysis

All data were charted in a form by an experienced dentist. All data were entered and analyzed using the IBM Corp. Released 2019. IBM SPSS Statistics for Windows, Version 28.0. Armonk, NY: IBM Corp. Categorical variables are presented as N (%) and continuous variables as Mean ± SD. Poisson regression was used to identify significant related variables for children's dft outcomes. The result is shown as an unadjusted rate ratio (RR) with a 95% confidence interval (CI) and the corresponding p-value. Factors significantly linked (p<0.20) from the bivariate analysis were included in the multivariate regression model for adjustment: age, adult oral hygiene, DMFT of adults, children's gender, family adaptability, and family cohesion. The outcome is presented as an adjusted rate ratio (RR), a 95% confidence interval (CI), and corresponding p-values. Variables are excluded from the final adjusted model if p>0.20 in bivariate analysis or p>0.05 in multivariate analysis. To ensure robustness and reproducibility, all statistical assumptions were assessed: normality of continuous variables, multicollinearity among independent variables, and homoscedasticity of residuals. A significance threshold of p < 0.05 was applied throughout.

## Results

Descriptive characteristics of participants

Thirty mother-child dyads from the Semai tribe participated, with mothers having a mean age of 35.7 years (SD 9.2). Children had a mean age of 7.1 years (SD 2.0), and slightly more than half were female. Over one-third of families demonstrated a disengaged type of cohesion, while nearly four out of five were classified as having a chaotic type of adaptability. Dental examinations revealed a high burden of disease: almost half of the children had caries in primary teeth (mean dft = 3.2), and more than 90% of mothers experienced caries (mean DMFT = 5.0). Only a small proportion of participants were caries-free. In terms of oral hygiene, more than half of the children were classified as poor. Table 1 shows the details of the participants' characteristics.

**Table 1:** Descriptive Characteristics of Orang Asli Children and Their Mothers (n = 30) Data are expressed as mean (± standard deviation) and N (%). N = Number of participants; % = Percentage; SD = Standard Deviation; dft = decayed and filled teeth in primary dentition; DMFT = decayed, missing, and filled teeth in permanent dentition.

Variables	N (%)	Mean (±SD)
Children		
Gender:		
Male	13 (43.3)	-
Female	17 (56.7)	-
Age (years):		
Male	-	8.38 (± 3.15)
Female	-	7.12 (± 1.97)
Caries level:		-
Caries free	16 (53.3)	-
Moderate caries	5 (16.7)	-
High caries	9 (30.0)	-
Prevalence of caries	14 (46.7)	-
Children’s mean dft	-	3.17 (± 4.94)
Oral Hygiene:		
Good	18 (60.0)	-
Fair	4 (13.3)	-
Poor	8 (26.7)	-
Mothers		
Age:	-	35.67 (±9.17)
18-29 years old	10 (33.3)	-
30-39 years old	7 (23.3)	-
40-49 years old	11 (36.7)	-
50-59 years old	2 (6.7)	-
Family Cohesion:		
Disengaged	11 (36.7)	-
Separated	5 (16.7)	-
Connected	10 (33.3)	-
Enmeshed	4 (13.3)	-
Family Adaptability:		
Rigid	2 (6.7)	-
Structured	4 (13.3)	-
Flexible	1 (3.3)	-
Chaotic	23 (76.7)	-
Caries level:		
Caries free	2 (6.7)	-
Moderate caries	8 (26.7)	-
High caries	20 (66.7)	-
Prevalence of caries	28 (93.3)	-
Mothers’ Mean DMFT	-	5.00 (± 4.19)
Oral Hygiene:		
Good	3 (10.0)	-
Fair	10 (33.3)	-
Poor	17 (56.7)	-

After the adjustments in the multivariate analysis, the following variables were predictors of caries in children: parents' age less than 40 years (RR=4.72; 95% CI: 1.02-21.9) and chaotic type of family adaptability (RR=6.85; 95% CI: 1.09-42.89). Results are shown in Table 2.

**Table 2:** Bivariate and Multivariate Analysis of Factors Associated with Children’s dft Scores RR = Rate Ratio; CI = Confidence Interval; df = degrees of freedom; SD = Standard Deviation; dft = decayed and filled teeth (primary dentition); DMFT = decayed, missing, and filled teeth (permanent dentition). “—” indicates reference category or not included in multivariate model. *Statistically significant at p < 0.05.

Variables	dft Children Mean (±SD)	Bivariate			Multivariate		
		Unadjusted Rate Ratio (RR)			Adjusted Rate Ratio (RR)		
		Wald Chi-Square (df)	95% CI	p-value	Wald Chi-Square (df)	95% CI	p-value
Age							
<40 years	5.06 (±5.89)	17.00 (1)	7.31 (2.84 – 18.81)	<0.001	3.926 (1.00)	4.72 (1.02 – 21.9)	0.048
≥40 years	0.69 (±1.03)	-	1.00	-	-	1.00	-
Adult Oral Hygiene							
Good	8.33 (±7.23)	4.879 (1)	3.46 (1.15 – 10.38)	0.027	-	-	-
Fair	2.90 (±5.43)	0.073 (1)	1.20 (0.32 – 4.56)	0.786	-	-	-
Poor	2.41 (±3.94)	-	1.00	-	-	-	-
DMFT mothers							
No caries	7.00 (±7.07)	4.109 (1)	3.89 (1.05 – 14.46)	0.043	-	-	-
Moderate	5.63 (±6.46)	3.842 (1)	3.13 (1.00 – 9.77)	0.049	-	-	-
High	1.80 (±3.64)	-	1.00	-	-	-	-
Children gender							
Female	3.35 (±4.09)	0.055 (1)	1.15 (0.36 – 3.61)	0.815	-	-	-
Male	2.92 (±5.20)	-	1.00	-	-	-	-
Family adaptability							
Rigid/structure/flex	0.29 (±0.49)	-	1.00	-	-	1.00	-
Chaotic	4.04 (±5.36)	16.326 (1)	14.15 (3.91-51.18)	<0.001	4.222 (1)	6.85 (1.09–42.89)	0.040
Family Cohesion							
Disengaged	3.91 (±5.72)	3.527 (1)	2.61 (0.96 – 7.08)	0.060	-	-	-
Separated	3.60 (±5.37)	1.746 (1)	2.40 (0.66 – 8.79)	0.186	-	-	-
Connected	2.80 (±5.20)	0.989 (1)	1.86 (0.55 – 6.39)	0.320	-	-	-
Enmeshed	1.50 (±1.00)	-	1.00	-	-	-	-

## Discussion

This pilot study identified two significant predictors of dental caries among Orang Asli children aged 5 to 12 years in Kampung Pelawan, Perak: mothers’ age and type of family adaptability. To our knowledge, this is among the first studies to examine family cohesion and adaptability as predictors of childhood caries in this community.

Caries status of Orang Asli children

The prevalence of primary tooth caries in this study was 46.7%, with a mean dft of 3.97. These figures are higher than the 41.4% prevalence and mean dft of 1.01 reported among OA children aged 11 to 12 years in a recent study [4]. The higher mean dft observed here may reflect an increased vulnerability to early childhood caries in younger children, potentially due to prolonged bottle feeding [25], higher intake of cariogenic foods [26], and limited access to preventive dental care [2]. These findings underscore the substantial burden of caries among OA children and the need for targeted early-life interventions.

Mothers’ age and children’s caries level

Mothers’ age emerged as a significant predictor of caries in this study. Younger OA mothers may encounter greater challenges in maintaining their children’s oral health due to less experience, lower oral health knowledge, or less stable household routines. Previous studies [27,28] have shown that socioeconomic challenges are more prevalent among younger mothers. These constraints may limit their ability to prioritize oral health care. The OA community is recognized as one of the most socioeconomically disadvantaged groups in Malaysia, and such constraints may further exacerbate caries risk. Additionally, individuals under 40 often belong to the “sandwich generation,” supporting both aging parents and young children [29,30]. This dual burden can increase stress and reduce the time and resources available for consistent oral health care. These factors may, in turn, worsen children’s caries outcomes [31].

Family cohesion, family adaptability, and caries

This study also found that “chaotic” family adaptability significantly contributed to higher caries levels in children. Chaotic adaptability, characterized by excessive flexibility and a lack of structure [17], may result in irregular oral health routines and reduced parental supervision [32], hence increasing caries level. Previous research has linked high family adaptability with lower oral health literacy (OHL) in children [10], which in turn has been associated with poorer oral health behaviors [33] and outcomes [34]. Interestingly, some studies have reported that higher OHL is associated with lower adaptability, particularly in “rigid” or “structured” families [35]. Differences in measurement methods may explain this discrepancy: our study assessed family adaptability from the mothers’ perspective, while others have relied on adolescent self-reports. Adolescents and parents often differ in their perceptions of family dynamics [36], suggesting that future research should collect perspectives from multiple family members to better understand these relationships.

In contrast, family cohesion did not show a significant direct association with children’s caries outcomes. This may be explained by the small sample size of this pilot study and the potential measurement limitations of FACES-III. Cultural dynamics in Orang Asli families, where extended kinship and collective caregiving are common, may also dilute the impact of immediate household cohesion. Cohesion may still exert an indirect influence, as suggested in other family health research, and should not be dismissed in future studies.

Public health and clinical implications

These findings highlight the importance of integrating family dynamics into oral health promotion. Families with stronger cohesion and appropriate adaptability often have better communication [37], healthier dietary patterns [9], and lower engagement in risk behaviors [38-40]. A family-centered approach, incorporating oral health education, parent-child toothbrushing routines, and shared goal setting, may improve children’s oral health outcomes. Evidence shows that family-based interventions are more effective than distributing educational materials alone [41]. Clinically, this approach can include dental screenings for the whole family, joint treatment planning, and brief counselling sessions to promote consistent oral hygiene practices at home [42]. Other than that, parental oral health education, structured parent-child toothbrushing routines, and caregiver-inclusive oral health promotion could also be implemented in the family-based intervention.

Among Orang Asli communities, collectivist values, intergenerational caregiving, and close community ties strongly shape child-rearing practices [43]. While these traditions provide valuable social support, they may also reduce parental autonomy in enforcing structured oral health routines, particularly among younger mothers with limited health literacy. Culturally tailored interventions should therefore involve extended caregivers and embed oral health promotion within familiar community practices. As this is a pilot study, these insights are preliminary, but they point toward the need for larger studies to design and evaluate family-based, culturally sensitive oral health programs for Indigenous populations.

Strengths and limitations

This study’s cross-sectional design precludes causal inference, as both exposure and outcome were assessed at a single time point. As a pilot study, the small sample size of 30 dyads limits statistical power and generalizability, which may also explain why family cohesion did not emerge as significant despite its theoretical importance. The regression models yielded low RR values and wide confidence intervals. Therefore, the findings should be interpreted cautiously and considered hypothesis-generating rather than definitive. Another limitation is the absence of child self-reports and oral health literacy measures. Relying solely on maternal reports may have restricted our ability to capture children’s own perspectives and the mediating role of oral health literacy in shaping oral health outcomes. Additionally, the study utilized FACES-III, which has since been superseded by FACES-IV. While FACES-III was widely validated and appropriate at the time of data collection, reliance on an older version may limit comparability with newer studies.

Despite these limitations, the study has several notable strengths. It is among the first to explore the influence of family cohesion and adaptability on oral health in an Indigenous Malaysian community, addressing a significant gap in the literature. Standardized clinical indices (DMFT/dft) enhanced methodological rigor. Importantly, this pilot also served as a cultural validation exercise. Feedback from Orang Asli mothers was systematically gathered and used to refine questionnaire terminology and contextualize abstract concepts, improving cultural fit and feasibility for the larger study. These strengths add value to the pilot by informing both methodological refinements and the design of culturally tailored interventions.

## Conclusions

This pilot study highlights the important role of family functioning and maternal factors in the oral health of Orang Asli children. Chaotic family adaptability, characterized by a lack of structure and inconsistent routines, was significantly associated with higher caries experience. Younger maternal age also emerged as a risk factor, underscoring the influence of parental characteristics on children’s oral health. While family cohesion and maternal oral health did not show direct significant associations, their potential indirect roles cannot be excluded and warrant further investigation. Future oral health promotion strategies should adopt a family-centered and culturally tailored approach, integrating parental education, caregiver support, and interventions that strengthen family adaptability while reinforcing cohesion. Larger studies across diverse Orang Asli subgroups are needed to confirm these associations and to inform the design of sustainable, family-based oral health interventions.

## Appendices

Appendix 1: Family Adaptability and Cohesion Evaluation Scale (FACES-III)

(Adapted from Olson DH, Portner J, Lavee Y. Family Adaptability and Cohesion Evaluation Scale III. St. Paul, MN: Family Social Science, University of Minnesota; 1985. Open access.)

Instructions (English): Please select the response that best describes your family now. Arahan (Bahasa Melayu): Sila pilih jawapan yang paling tepat menggambarkan keadaan keluarga anda sekarang.

Response scale / Skala jawapan: 1 = Almost Never / Hampir Tidak Pernah 2 = Once in a While / Sekali-sekala 3 = Sometimes / Kadang-kadang 4 = Frequently / Kerap 5 = Almost Always / Hampir Sentiasa

**Table 3:** Family Adaptability and Cohesion Evaluation Scale (FACES-III) in English and Malay

No.	English Version	Malay Version Asked to the Participants
	Family members ask each other for help.	Ahli-ahli keluarga anda saling membantu
	In solving problems, the children’s suggestions are followed.	Dalam menyelesaikan masalah keluarga, cadangan daripada anak-anak dipertimbangkan/ diikuti dalam penyelesaian masalah
	We approve of each other’s friends.	Anda dan anak-anak saling menerima kawan masing-masing
	Children have a say in their discipline.	Anak-anak dalam keluarga anda diberikan ruang untuk bersuara mengenai disiplin mereka
	We like to do things with just our immediate family.	Anda dan keluarga anda suka melakukan sesuatu hanya dengan ahli keluarga terdekat
	Different persons act as leaders in our family.	Ada beberapa orang dalam keluarga anda yang bertindak sebagai pemimpin dalam keluarga kami.
	Family members feel closer to other family members than to people outside the family.	Ahli keluarga anda berasa lebih rapat antara satu sama lain berbanding dengan orang yang bukan ahli keluarga
	Our family changes its way of handling tasks.	Keluarga anda mengubah cara tentang sesuatu tugasan itu dikendalikan jika perlu
	Family members like to spend free time with each other.	Ahli keluarga anda suka meluangkan masa lapang antara satu sama lain
	Parent(s) and children discuss punishment together.	Ibu bapa dan anak-anak berbincang bersama tentang hukuman atau tindakan disiplin
	Family members feel very close to each other.	Ahli keluarga anda berasa sangat rapat antara satu sama lain.
	The children make the decisions in our family.	Anak-anak dalam keluarga anda diberi peluang untuk membuat keputusan dalam keluarga
	When our family gets together for activities, everybody is present.	Apabila keluarga anda berkumpul untuk melakukan aktiviti, semua ahli keluarga akan hadir.
	Rules change in our family.	Peraturan boleh berubah dalam keluarga anda
	We can easily think of things to do together as a family.	Anda dan ahli keluarga boleh dengan mudah memikirkan perkara yang boleh dilakukan bersama sebagai sebuah keluarga
	We shift household responsibilities from person to person.	Kami memindahkan tanggungjawab menjaga rumah dari sesama kami
	Family members consult other family members on their decisions.	Ahli keluarga anda berunding sesama sendiri mengenai keputusan yang telah diambil.
	It is hard to identify the leader(s) in our family.	Sukar untuk mengenal pasti siapakah ketua (atau ketua-ketua) dalam keluarga anda
	Family togetherness is very important.	Kebersamaan dalam keluarga itu amat penting.
	It is hard to tell who does which household chores	Sukar untuk menentukan siapa yang melakukan sesuatu kerja
No.	English Version	Malay Version Asked to the Participants
	Family members ask each other for help.	Ahli-ahli keluarga anda saling membantu
	In solving problems, the children’s suggestions are followed.	Dalam menyelesaikan masalah keluarga, cadangan daripada anak-anak dipertimbangkan/ diikuti dalam penyelesaian masalah
	We approve of each other’s friends.	Anda dan anak-anak saling menerima kawan masing-masing
	Children have a say in their discipline.	Anak-anak dalam keluarga anda diberikan ruang untuk bersuara mengenai disiplin mereka
	We like to do things with just our immediate family.	Anda dan keluarga anda suka melakukan sesuatu hanya dengan ahli keluarga terdekat
	Different persons act as leaders in our family.	Ada beberapa orang dalam keluarga anda yang bertindak sebagai pemimpin dalam keluarga kami.
	Family members feel closer to other family members than to people outside the family.	Ahli keluarga anda berasa lebih rapat antara satu sama lain berbanding dengan orang yang bukan ahli keluarga
	Our family changes its way of handling tasks.	Keluarga anda mengubah cara tentang sesuatu tugasan itu dikendalikan jika perlu
	Family members like to spend free time with each other.	Ahli keluarga anda suka meluangkan masa lapang antara satu sama lain
	Parent(s) and children discuss punishment together.	Ibu bapa dan anak-anak berbincang bersama tentang hukuman atau tindakan disiplin
	Family members feel very close to each other.	Ahli keluarga anda berasa sangat rapat antara satu sama lain.
	The children make the decisions in our family.	Anak-anak dalam keluarga anda diberi peluang untuk membuat keputusan dalam keluarga
	When our family gets together for activities, everybody is present.	Apabila keluarga anda berkumpul untuk melakukan aktiviti, semua ahli keluarga akan hadir.
	Rules change in our family.	Peraturan boleh berubah dalam keluarga anda
	We can easily think of things to do together as a family.	Anda dan ahli keluarga boleh dengan mudah memikirkan perkara yang boleh dilakukan bersama sebagai sebuah keluarga
	We shift household responsibilities from person to person.	Kami memindahkan tanggungjawab menjaga rumah dari sesama kami
	Family members consult other family members on their decisions.	Ahli keluarga anda berunding sesama sendiri mengenai keputusan yang telah diambil.
	It is hard to identify the leader(s) in our family.	Sukar untuk mengenal pasti siapakah ketua (atau ketua-ketua) dalam keluarga anda
	Family togetherness is very important.	Kebersamaan dalam keluarga itu amat penting.
	It is hard to tell who does which household chores	Sukar untuk menentukan siapa yang melakukan sesuatu kerja
